# Insights into the Role of the Habenular Circadian Clock in Addiction

**DOI:** 10.3389/fpsyt.2015.00179

**Published:** 2016-01-05

**Authors:** Nora L. Salaberry, Jorge Mendoza

**Affiliations:** ^1^CNRS UPR-3212, Institute of Cellular and Integrative Neurosciences, University of Strasbourg, Strasbourg, France

**Keywords:** circadian system, habenula, drug of abuse, addiction, dopamine

## Abstract

Drug addiction is a brain disease involving alterations in anatomy and functional neural communication. Drug intake and toxicity show daily rhythms in both humans and rodents. Evidence concerning the role of clock genes in drug intake has been previously reported. However, the implication of a timekeeping brain locus is much less known. The epithalamic lateral habenula (LHb) is now emerging as a key nucleus in drug intake and addiction. This brain structure modulates the activity of dopaminergic neurons from the ventral tegmental area, a central part of the reward system. Moreover, the LHb has circadian properties: LHb cellular activity (i.e., firing rate and clock genes expression) oscillates in a 24-h range, and the nucleus is affected by photic stimulation and has anatomical connections with the main circadian pacemaker, the suprachiasmatic nucleus. Here, we describe the current insights on the role of the LHb as a circadian oscillator and its possible implications on the rhythmic regulation of the dopaminergic activity and drug intake. These data could inspire new strategies to treat drug addiction, considering circadian timing as a principal factor.

## The Circadian System

Environmental signals are rhythmic and organisms have to adapt to daily changes imposed by the day–night alternation. Species have, therefore, developed timekeeping mechanisms that are regulated by circadian (*circa* = close to, *dien* = day) clocks in almost any cell. These clocks are able to oscillate in a self-sustained manner with a periodicity close to a day (24 h), and to transmit time information to the rest of the body through specific output pathways (1).

The mammalian hypothalamus contains the principal circadian clock in the suprachiasmatic nucleus [SCN; (2)], which controls most of the behavioral and physiological rhythms (e.g., locomotor activity, hormone secretion). In every SCN cell, the clockwork is dependent on an oscillatory mechanism formed from transcription-translational feedback loops in which the expression of clock genes like, *Clock*, *Bmal1*, *Per (1–3)*, *Cry (1–2)*, and *Rev-erb*α, and their respective proteins, runs around 24 h (3).

Light is the most important synchronizer for the SCN, this relays on a direct pathway from the photosensitive retinal ganglion cells that use the photopigment melanopsin (4). Besides light stimulation, non-photic time cues, such as exercise, food restriction, or drug intake, are able to affect or synchronize the SCN clock activity as well (5–7).

## The Clock on Drugs

In humans and animals, drug intake often results in disruptions of daily rhythms [e.g., locomotor activity, sleep–wake cycle, eating habits; (5, 8)]. In rodents, several drugs of abuse like methamphetamine, cocaine, ethanol, and morphine affect the period of circadian rhythms of behavior and physiology [e.g., locomotion, body temperature (5, 8, 9)].

Furthermore, people with perturbations of the circadian system [e.g., shift-workers, people experiencing frequent jet-lags; (10)], or animal models with disruptions of the body clock [e.g., chronic jet-lag exposure; (11)] show an increase of drug intake (e.g., psychostimulants). For example, shift-workers use methamphetamine to avoid sleepiness and perform tasks accurately during their working hours. Methamphetamine intake occurs mainly at the beginning of the active phase regardless of the day–night period (10), suggesting that drug intake may have a synchronizer role for the circadian clock (8).

Reciprocally, the circadian system modulates the behavioral responses to drug intake. In some clinical reports drug overdoses and toxicity have been showed to be clock-dependent (12–14). In rodents, behavioral sensitization and reward responses (measured by a conditioned place preference, CPP) to chronic injections of cocaine are significantly higher when animals are treated in the early day than at dusk or night (15, 16). Furthermore, some studies showed that the hormone melatonin, which is secreted at night, modulates the day–night variations in cocaine sensitization and CPP in mice (16–19).

In rats, cocaine self-administration or alcohol intake shows a daily rhythm with higher consumption during the night (20–22). Nonetheless, for cocaine, daily rhythms are observed only when access is restricted to two or three intakes per hour. Over three intakes/h drug intake increases and the rhythm is disrupted (20). Interestingly, under constant darkness conditions, a circadian rhythm of cocaine intake emerges with a higher consumption at night, indicating that an endogenous clock regulates the circadian activity of cocaine intake (23).

Circadian genes from the molecular clockwork regulate drug intake and drug-related behaviors. Mutations of the genes *Per2* or *Clock* (e.g., *Clock*^Δ^*^*19*^* mice) lead to higher behavioral responses (e.g., CPP and behavioral sensitization) to chronic injections of cocaine, and an increase in alcohol consumption (15, 24–26). Interestingly, animals lacking the gene *Per1* or *Npas2* (a homolog of the *Clock* gene) are behaviorally less sensitive to cocaine (15, 27), showing opposite effects to drug intake to those observed in *Per2* and *Clock* mutants. Together, these data suggest an important link between drug intake, the circadian system, and the master clock in the SCN.

Although the SCN is the principal pacemaker, other circadian oscillators in the brain and peripheral organs are present (28). More importantly, in brain structures regulating reward and behavior, circadian oscillations with a similar molecular machinery to the SCN have been reported (28–30). This suggests that, beyond the SCN, a multi-oscillatory circadian system in the mammalian brain composed of the reward centers, *inter alia*, may regulate behavior and drug intake (31).

## The Habenula

The habenula (Hb) is a brain region, which with the pineal gland forms the epithalamus. It is an interesting structure involved in the control of behavior (32). The Hb is located dorsally along the third ventricle close to the dorso-medial thalamus. It is mainly divided into two regions: the median Hb (MHb) and the lateral Hb (LHb) nuclei. Both regions receive forebrain information via the fibers of the *stria medullaris* and project to the midbrain via the efferent fibers of the *fasciculus retroflexus* (FR). The MHb and LHb contain sub-nuclei that differ in their receptors, cell morphology, anatomical and functional inputs and outputs, and neurotransmitters (33–36).

The LHb is a relay structure and, according to Hikosaka (37), its role is mainly related to the suppression of motor activity. However, several studies showed that the LHb is involved in other brain functions, such as reward, aversion, cognition, maternal behavior, sleep, and circadian rhythms, and in brain dysfunctions, such as addiction, depression, and schizophrenia (35–38).

### The LHb in the Dopamine Circuitry: Implications in Addiction

**Figure 1:**
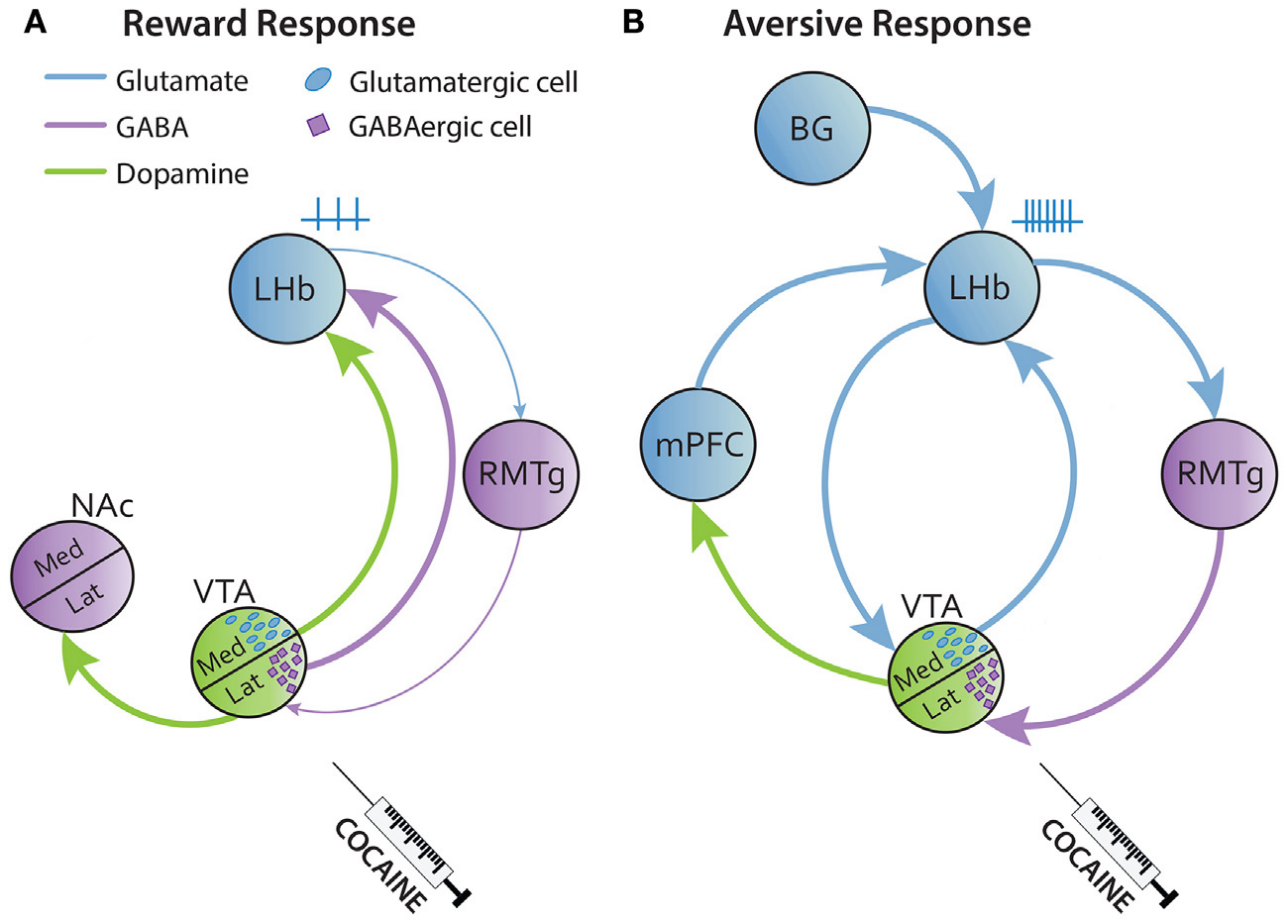
**Simplified circuitry of reward and aversive responses of the LHb to drug intake**. **(A)** Acute injections of cocaine activate the lateral VTA inducing DA release to the lateral NAc (shell) leading in a reward response. The LHb receives GABAergic and DAergic fibers from the VTA that inhibit the LHb. **(B)** Cocaine also has a delayed aversive effect that follows the reward response. Here, the LHb is activated by several glutamatergic excitatory inputs from BG, mPFC, and medial VTA that will be facilitated by cocaine. BG, basal ganglia; Med, median; mPFC, medial prefrontal cortex; Lat, lateral.

The reward system is an intricate network involving diverse structures. The keystone of this circuit is the communication between the ventral tegmental area (VTA) and the nucleus accumbens (NAc). Dopaminergic neurons of the lateral VTA, which project to the lateral part of the NAc, encode for reward or for aversion depending on the dopamine (DA) receptors involved (39). Few studies reported NAc projections to LHb cells (40), although this has to be confirmed. Furthermore, the medial prefrontal cortex (mPFC) receives DAergic fibers from the medial VTA and sends glutamatergic projections to the NAc and LHb [(36, 39, 41); Figure 1].

The VTA is known to be a DAergic source, although some VTA neurons also contain GABA and glutamate. In this regard, some studies reported that the LHb is innervated by DAergic fibers and also by glutamate and GABAergic projections from the medial and lateral part of the VTA, respectively (42–45). In humans, there is a functional correlation between the LHb and VTA during aversion process, indicating a strong coupling of these structures (46). Thus, VTA-GABAergic transmission to the LHb may encode reward, while glutamatergic transmission results in aversion [(47); Figures 1A,B].

LHb neurons, which are mainly glutamatergic, are activated by an aversive cue, an unrewarding task or an error of prediction (i.e., absence of expected reward), and they are inhibited by an expected or unexpected reward (32, 48). Interestingly, DAergic VTA neurons behave the other way round in the presence of a reward or an aversive stimulus (32, 49, 50). Glutamatergic LHb projections terminate mostly on the rostromedial tegmentum nucleus (RMTg), which is a GABAergic nucleus inhibiting the lateral VTA, which then projects to the NAc (41, 51). Thus, the LHb–RMTg–VTA circuit predicts appetitive or aversive outcomes. There are also few LHb glutamatergic fibers directly innervating DAergic neurons of the medial VTA which modulate the activity of the mPFC to drive aversion [(41, 43, 51); Figures 1A,B].

Cocaine intake has an initial rewarding effect, which depends on the DAergic circuit to the NAc, which is followed by a negative effect in which glutamate might play an important role. Due to the close functional relationship of the LHb with the DAergic reward system, its implication in positive and negative effects of drug intake has been extensively studied. The LHb is a structure very sensitive to cocaine. A low dose of cocaine increases the glucose uptake in this region in rats (52). *In vivo* and *in vitro* cocaine exposure leads to an inhibitory (reward effect) or a biphasic firing rate response of the LHb. This LHb biphasic response (inhibitory then an excitatory effect) represents the reward and aversive states after cocaine intake, respectively [(53); Figures 1A,B]. Moreover, cocaine induces an increase of AMPA receptors in the LHb that facilitates glutamatergic inputs (from the VTA, basal ganglia, or mPFC), leading to LHb hyperactivity and hyperexcitability (Figure 1B). This LHb hyperactivity might have a feedback role to prevent a higher activation of the VTA [(36, 43, 54–59); Figure 1B].

The role of the LHb in drug intake has also been highlighted in studies using lesions. LHb-ablated animals do not extinct drug-seeking behavior while cocaine intake is not affected, suggesting that animals are not able to decrease drug-seeking even if there are no more rewarding effects (60). Voluntary ethanol intake is higher in rats with LHb lesions than that in sham control animals (61). In sum, these results show the important role of the LHb in the regulation of drug-directed behaviors and in the mediation of drug intake effects.

The MHb seems to play a role in the regulation of behavior and drug addiction as well. Mainly the MHb has been implicated in nicotine addiction (62). However, in mice with lesions of the dorsal part of the MHb, voluntary motor activity and sucrose preference are affected, suggesting an important role of the MHb in diverse motivated behaviors (63). Moreover, MHb inhibition by the antagonists of nicotinic receptors or optogenetic manipulations blocks drug self-administration (e.g., cocaine, morphine, alcohol) or leads to an aversive response, respectively (62, 63).

Hence, habenular nuclei (LHb and MHb) may have different impact on behavior and drug intake. This difference might depend on their particular function, the circadian properties of each structure (i.e., period, phase, and amplitude), and/or the time of drug exposure.

### The Circadian Clock in the Lateral Habenula

**Figure 2:**
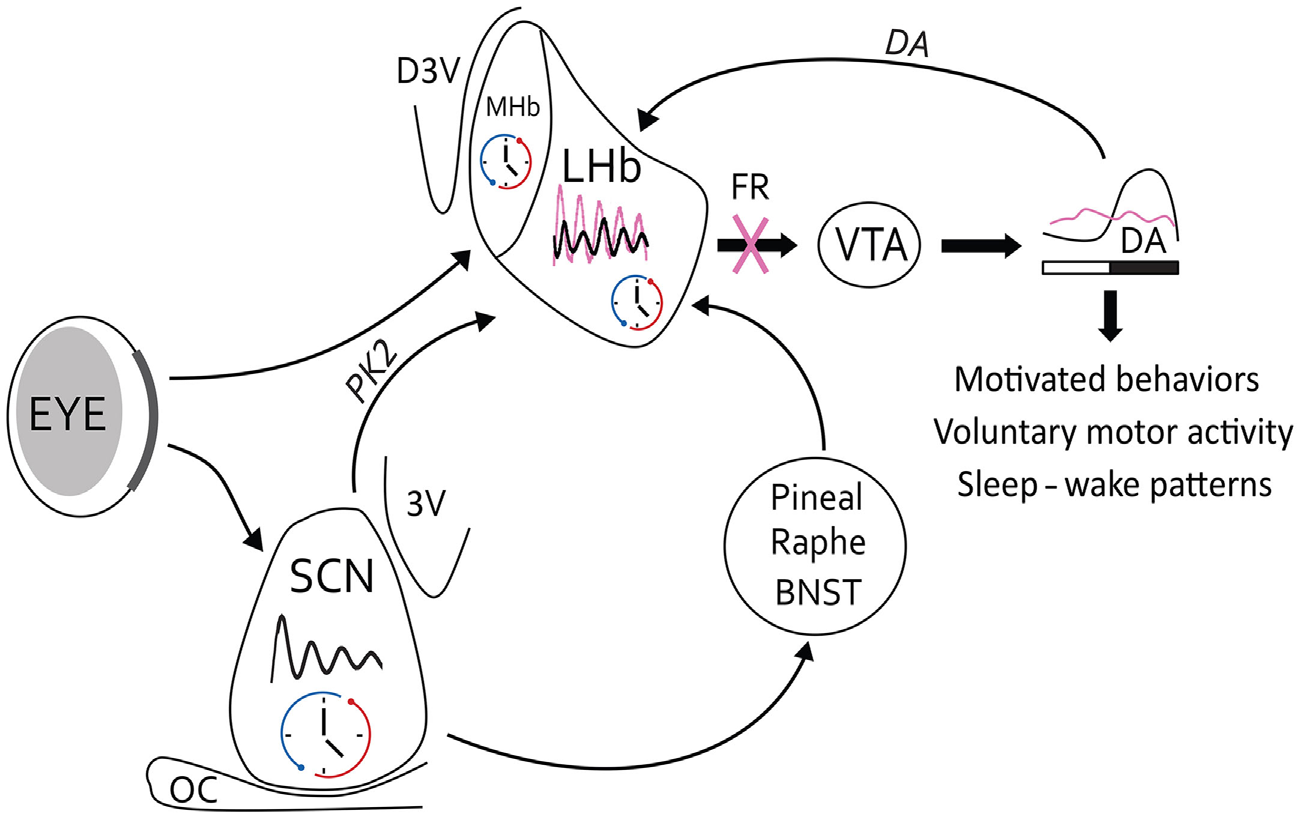
**Circadian circuitry of LHb for the control of behavior**. The circadian oscillator in the LHb may be affected by light information from retinal projections (in bold), which arrive at the border of the LHb. The main SCN clock contacts the LHb by a direct neural pathway in which PK2 is positioned as the main clock-output factor. However, possible indirect pathways via the pineal gland, raphe nuclei, or BNST may link the SCN to the LHb as well. The LHB modulates midbrain DA activity from the VTA, and reciprocally DA may affect LHb activity. Under normal conditions (black oscillation), this pathway may result in a circadian release of DA in the striatum and several behavioral outputs, such as motivated behavior, voluntary motor activity, or sleep–wake cycles. On the other hand, under acute cocaine conditions, circadian rhythms of the LHb may change in amplitude, phase, or period (pink oscillations), which can lead in modifications of the VTA activity. However, under chronic cocaine situations beyond changes in LHb oscillations, denervations of the FR (pink cross) may lead to a dysregulation in the DAergic system and related behavioral outputs. Thus, symptoms of addictive behaviors may appear due to loss of rhythmic control of LHb. 3V, ventral third ventricule; BNST, bed nucleus of the stria terminalis; D3V, dorsal third ventricule; OC, optic chiasma.

Beyond the role of the Hb on behavioral control, recent data have shown an implication of the Hb in the regulation of sleep and circadian rhythms. Electrophysiological recordings showed that both LHb and MHb cells present a circadian variation of their spontaneous firing rate [(64–67); Figure 2]. This rhythmic activity disappears in both the MHb and LHb in animals with a molecular circadian clock mutation (65, 66).

Other studies report the expression of *Per1–2* and *Clock* mRNA and protein in the whole Hb complex (68, 69). Using transgenic mice in which a luciferase reporter is coupled to the protein PER2, authors showed that the expression of PER2 in a small selected part in the mid-LHb has a sustained rhythmic activity (64). Moreover, c-Fos expression in the LHb is also rhythmic in mice, hamsters, and rats (70). Interestingly, denervations of the FR, the main output of the LHb, alter the circadian rest-activity cycle in hamsters (71). Thus, the LHb clock modulates the intensity or amount of activity in the day–night rhythms of locomotion.

The LHb receives environmental light information from melanopsin ganglion cells of the retina that project to the border of the LHb; thus, some interneurons may link the retino-LHb pathway [(72); Figure 2]. In addition, firing rate of LHb cells is affected by light *in vivo*, and this photic response is significantly larger in amplitude in LHb cells during the night than during the day (67).

The SCN clock innervates the LHb (Figure 2). The SCN uses different neuropeptide, such as vasopressin, vasoactive intestinal peptide, and prokineticin 2 (PK2), to communicate time information to other brain structures (73). The LHb receives PK2 fibers from the SCN and contains PK2 receptors [(74); Figure 2]. A recent study reported that PK2 is able to inhibit the LHb firing rate *in vitro* (66). Some vasopressin fibers run around the ventral part of the LHb even in SCN-lesioned rats, suggesting that vasopressin source might come from other brain structure (75); the bed nucleus of the stria terminalis, or the paraventricular nucleus of the hypothalamus (76) for example.

An indirect pathway can drive the message from the SCN to the LHb by a hormonal input (e.g., melatonin), or by a neuronal pathway with an intermediate structure, such as the raphe nuclei that project to both the SCN and LHb [(77–79); Figure 2]. Interestingly, serotonin release from the raphe nuclei is also rhythmic; it modulates SCN activity and dampens excitatory inputs from the basal ganglia to the LHb (54, 80, 81). Thus, serotonin may be a good candidate to modulate the rhythmic activity of the LHb. Reciprocally, the LHb projects to the raphe nuclei (82), and drugs of abuse act on serotonin transporters (83). Serotonin levels are altered in depression and mood signs are often associated symptoms of addicted behaviors. Therefore, the mood changes during addiction may be dependent on disturbance in the circadian link between the LHb and the raphe nuclei (84).

### What May Be the Clock-Outputs of the LHb to Control Rhythmic Drug Intake?

The VTA expresses clock genes and tyrosine hydroxylase (the limiting enzyme in DA synthesis) co-localizes with clock proteins [REV-ERB, BMAL1, PER1; (85–87)]. DA release and cell firing in the VTA are rhythmic (88–90). Moreover, the PER2 protein expression in the striatum follows a circadian rhythm (peak of expression rises at night) that is entrained by DA via D2 type receptors (91). However, in isolated cultured VTA from *Per1: Luciferase* rats, no oscillations were found (92). Thus, VTA circadian oscillations must be under the control of a brain self-sustained circadian oscillator.

Day–night differences of tyrosine hydroxylase and DA transporter in striatum are dampened but not abolished in SCN-lesioned rats (93). Therefore, another circadian clock beyond the SCN regulates rhythmic activity of the DAergic system. The LHb clock could play an important role for the regulation of DAergic rhythms (Figure 2).

The circadian role of the LHb might be to inhibit DAergic neurons at a specific time during the 24-h cycle. In rats, the LHb peak of electrical activity occurs during the day, when the release of DA to the striatum is low (67, 91). In mice, however, there is a circadian rhythm of LHb firing rate with a peak at night time, which correlates with VTA lower electrical activity (64, 94). Thus, it is necessary to determine the circadian activity (i.e., firing rate, clock gene expression) of the LHb *in vivo* in both rats and mice.

Since LHb activity controls DA neuronal activity, it is also possible that LHb modulates rhythmic behaviors related to drug intake through the regulation of the DAergic system. In fact, behavioral sensitization [which has a rhythmic profile; (15, 16)] is altered by LHb lesions in rats (95). This suggests that the LHb clock could modulate the rhythmic patterns of behavioral sensitization or reward effects of cocaine. Moreover, cocaine self-administration shows a daily rhythm in phase (with a maximum at the night) with the peak of DA release in the striatum. Both self-administration and DA release are correlated with the highest LHb firing rate (20, 23, 67, 91). Importantly, chronic intake of cocaine, which leads to FR degeneration, is able to disrupt circadian rhythms probably due to the lack of LHb control on monoamine nuclei [(20, 96); Figure 2].

### Possible Therapies for Addiction: The Habenula as a Target

Low frequency stimulations, which mimic inputs from the VTA or raphe nucleus to the LHb, inhibit the LHb activity and induce cocaine intake in rats (35–38, 60). However, high frequencies, which mimic aversive and excitatory input from the basal ganglia, do not reduce cocaine intake (35–38, 60). Interestingly, deep brain stimulation (DBS) is associated with reward and aversive inputs (low and high frequencies), and reduces cocaine consumption in rats (60). DBS is a technical approach that has been used for the treatment of some psychiatric disorders, such as depression and obsessive–compulsive disorder (97).

The LHb has been proposed as a target for DBS to treat addiction because of its close functional relationship with the reward system (98). However, chronic exposure to cocaine, amphetamine, methamphetamine, MDMA, or nicotine results in the degeneration of the FR, which blocks the effect of DBS on drug intake (96, 99, 100). As long as FR is able to transmit information, the LHb can be considered as a main target for DBS in the treatment of addiction. Moreover, if circadian properties of the LHb are an important factor for DBS, it then becomes possible to potentiate the beneficial effect, or avoid side effects of DBS, by applying stimulation at a specific time of the day and consider a genuine type of DBS chronotherapy (i.e., time-dependent treatment).

Despite the prominent actions of DBS as a treatment, the technique still remains an invasive approach that may produce side effects. Therefore, other alternative treatments for addiction have to be considered.

In chronic drug intake, degeneration of FR may be due, in part, to a reduction of the GABA receptors expression, leading to a LHb disinhibition, and an increase of glutamate receptors allowing an excitotoxic effect on LHb cells (58, 96, 99). LHb disinhibition may be restored by a GABA_B_ receptor agonist, a molecule that is already proposed as a possible treatment for drug addiction (101). Hence, LHb inhibition by GABA agonists at the circadian optimal time could rescue a daily rhythm of LHb activity and reduce addiction states.

The Hb expresses melatonin receptors (102) and projects to the pineal gland, the site of melatonin production (103). Melatonin receptors mediate the behavioral sensitization to methamphetamine (104). Moreover, activation of melatonin receptors (with melatonin or the agonist agomelatine) reduces relapse-like alcohol intake in rats (105). More importantly, since there is a strong rhythmic activity of melatonin, it is possible that the effects of the hormone on the circadian activity of the Hb are phase (time)-dependent. Thus, melatonin might have effects to modulate drug-seeking and intake via an activation of its receptors in the Hb at specific times of the day.

## Conclusion

In this review, we addressed the circadian relevance of the LHb for the regulation of rhythmic brain activity and behavior. This work may help to understand the role of LHb, as a circadian clock, in the development of psychiatric pathologies and addiction. The vast literature devoted to LHb indicates that these nuclei have a relevant role in different brain functions, and dysfunctions (e.g., depression and addiction) (106). Some studies also highlight the clock properties of the LHb in the regulation of behavior (70, 71, 107). Therefore, to understand the specific neurobiological role of the Hb (both the MHb and LHb nuclei) in brain physiology and pathophysiological conditions, the circadian properties of this brain locus should be considered as an important factor.

## Conflict of Interest Statement

The authors declare that the research was conducted in the absence of any commercial or financial relationships that could be construed as a potential conflict of interest.

## Abbreviations

CPP, conditioned preference place; DA, dopamine; DBS, deep brain stimulation; FR, *fasciculus retroflexus*; Hb, habenula; LHb, lateral habenula; MHb, median habenula; mPFC, medial prefrontal cortex; NAc, nucleus accumbens; PK2, prokineticine2; RMTg, rostromedian tegmentum nucleus; SCN, suprachiasmatic nucleus; VTA, ventral tegmental area.
